# The complete mitogenome dataset of the Critically Endangered estuarine pipefish, *Syngnathus watermeyeri*

**DOI:** 10.1016/j.dib.2023.109864

**Published:** 2023-11-29

**Authors:** Arsalan Emami-Khoyi

**Affiliations:** Centre for Ecological Genomics and Wildlife Conservation^1^, University of Johannesburg, Auckland Park 2006, South Africa

**Keywords:** Mitogenome, Critically Endangered species, estuarine pipefish, *Syngnathus watermeyeri*, Divergence time

## Abstract

The Critically Endangered South African estuarine pipefish, *Syngnathus watermeyeri*, is one of the rarest teleost fish on the planet. In this analysed dataset, the complete mitochondrial genome of this species was assembled, annotated, and described. In addition, its evolutionary history was reconstructed in a Maximum Likelihood and a Bayesian framework. A circular mitochondrial contig 16 449 bp in length was assembled. A total of 13 protein-coding genes, 22 tRNAs and two rRNAs were annotated. The mitochondrial phylogenetic analysis showed that *S. watermeyeri* diverged from its widely distributed southern African sister species *S. temminckii* approximately 1.5 million years ago, and the ancestor of these two southern Afican pipefish species diverged from a clade of northern hemisphere pipefishes comprising *S. acus, S. rostellatus,* and *S. typhle* approximately 5.3 million years ago. The dataset presented here serves as the first step in understanding the evolutionary history of Africa's rarest pipefish.

Specifications Table

**Table:** 

Subject	Genomics
Specific subject area	Mitogenomics, Critically Endangered pipefish, *Syngnathus watermeyeri*, Bayesian phylogenetics, Maximum Likelihood, divergence
Data format	Raw and Analyzed
Type of data	Figures: Mitogenome circular map, amino acid frequency, codon skew, synonymous codon usage, dated phylogenetic tree. Table: List of Syngnathidae mitogenomes used for phylogenetic study, Mitogenome annotation feature table. FASTA: Mitogenome sequence data
Data collection	Tissue collection: Fin-clip biopsy Genomic DNA extraction: Qiagen DNeasy Blood & Tissue Kit (Qiagen, Hilden, Germany) Genomic library preparation: Diagenode Bioruptor (Diagenode, New Jersey, USA), NOVOKit (Novogene, Beijing, PRC), NovaSeq 6000 SP platform (Illumina, San Diego, USA). Quality check: FastQC v.0.12, Trimmomatic v.3.9. Mitogenome assembly and annotation: NOVOPlasty v.4.3, GetOrganelle v.1.7, MITOS webserver, MitoZ v.3.6. Phylogenetic analysis: BEAST2 v.2.5, RB package, Tracer 1.7. Phylogenetic tree visualisation: Figtree v.1.4, Chloroplot v.0.2, strap v.1.6
Data source location	Institute: University of Johannesburg City: Johannesburg Province: Gauteng Country: South Africa Latitude and longitude of the specimen collection site: (−33.67378 south, 26.67484 east) Email: molzoo@uj.ac.za
Data accessibility	Repository name: NCBI nucleotide database Data identification number: OR496150 Direct URL to data: https://www.ncbi.nlm.nih.gov/nuccore/OR496150.1/ Repository name: NCBI BioProject Data identification number: PRJNA1007887 Direct URL to data: https://www.ncbi.nlm.nih.gov/bioproject/PRJNA1007887/ Repository name: NCBI BioSample Data identification number: SAMN37105227 Direct URL to data: https://www.ncbi.nlm.nih.gov/biosample?Db=biosample&DbFrom=bioproject&Cmd=Link&LinkName=bioproject_biosample&LinkReadableName=BioSample&ordinalpos=1&IdsFromResult=1007887 Repository name: Sequence Read Archive Data identification number: SRR25722163 Direct URL to data: https://trace.ncbi.nlm.nih.gov/Traces/?view=run_browser&acc=SRR25722163&display=metadata Repository name: The African Centre of DNA Barcoding DNA Bank, University of Johannesburg (https://www.acdb.co.za/dna-bank) Data identification number: ID= 13,607, Collecting number = E6KMY, Museum Voucher ID= AEK001, DNA bank number= UJ13607 Direct URL to data: (Downloadable record) https://www.acdb.co.za/s/UJ-DNA-Bank-Database‑oct-2023.xlsx
Related research article	NA

## 1 Value of the Data

• The mitogenomic data presented here provides the first complete mitochondrial genome of *Syngnathus watermeyeri*, a rare pipefish species from South Africa.
• It contributes towards understanding the antitropical distributions of two endemic African species (*S. watermeyeri and S. temminckii*) and their European sister species.
• The dataset can also contribute towards studying the placement of the genus *Sygnathus* among other members of the bony fish family Syngnathidae, which includes pipefishes, seahorses and seadragons.
• The dataset can help in the establishment of effective management plans to preserve genetic diversity in the last remaining populations of the Critically Endangered estuarine pipefish.

## 2 Background

**Fig. 1:**
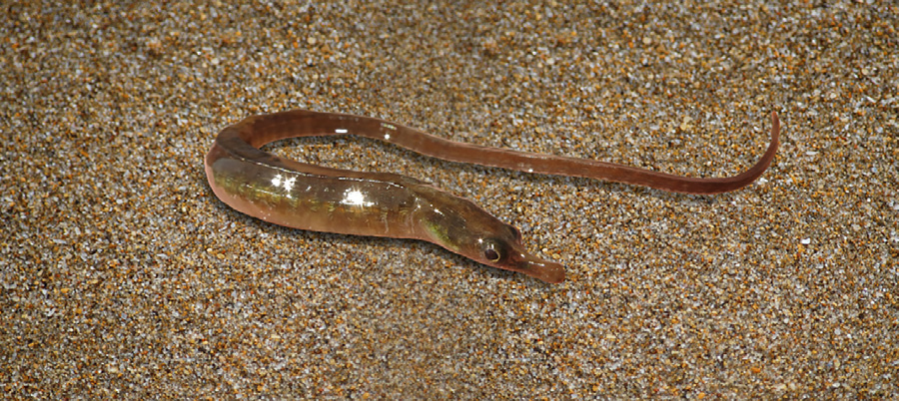
A photo of the Critically Endangered estuarine pipefish, *Syngnathus watermeyeri* (source=https://commons.wikimedia.org/wiki/File:Syngnathus_watermeyeri.png).

The southeast coast of South Africa is the last refuge of *Syngnathus watermeyeri* Smith, 1963*,* commonly known as the “estuarine pipefish” (Fig. 1). This pipefish is one of the rarest estuarine fish species on the planet, so rare that in 1994, it was listed as extinct by the IUCN Red List of Threatened Animals, after surveys of the estuarine habitats in its historical range that were conducted between 1989 and 1992 failed to record a single specimen [1]. After years of presumed extinction, a small population was discovered in the East Kleinemonde, an estuary where it had not previously been recorded. In 2003, a freshwater flood destroyed the seagrass habitats in this estuary, resulting in the local extinction of the estuarine pipefish. In 2007, a survey of estuarine ichthyofauna discovered a small cohort of juvenile fish in its historical home range, the Kariega Estuary. Subsequently, the species was also found in the adjacent Bushman's Estuary [2]. A genome-wide population study of *S. watermeyeri* in its remaining habitats revealed that both populations are inbred and do not show genetic differentiation [3], highlighting the urgency of understanding the evolutionary history of this species.

The preferred habitat of the estuarine pipefish is submerged macrophytes with a moderate current that can facilitate their predation on small invertebrate prey. Similar to other species of pipefish, it has low fecundity, poor swimming capability and small home ranges, making it particularly vulnerable to alterations in their estuarine habitat [4,5].

There are only two species of pipefish in the estuaries on South Africa's south coast: the longsnout pipefish *Syngnathus temminckii* Kaup, 1856 and the estuarine pipefish. These two species are readily distinguishable morphologically, with the snout being distinctly shorter in *S. watermeyeri*, and the body shape shorter and broader [4,5].

Why its sister species is common, whereas *S. watermeyeri* is on the brink of extinction and has a very small distribution range, is an interesting question. In the absence of a fossil record that could explain changes in the historical ranges of the two that would point to the replacement of the rare species by the common one, molecular methods are the only tool available to explore this issue.

Mitochondrial DNA (mtDNA) is the marker of choice to reconstruct the evolutionary history of wild species. While a single mitochondrial marker might not have enough information to conclusively resolve complex taxonomic relationships, the level of information contained in the complete mitogenome is typically sufficient to reconstruct phylogenetic relationships with high confidence.

The current dataset is the first assembled and annotated complete mitogenome of *S. watermeyeri*, and this information was used, for the first time, to estimate the divergence time between this species and its closely related congeners in the southern and northern hemispheres.

## 3 Data Description

**Fig. 2:**
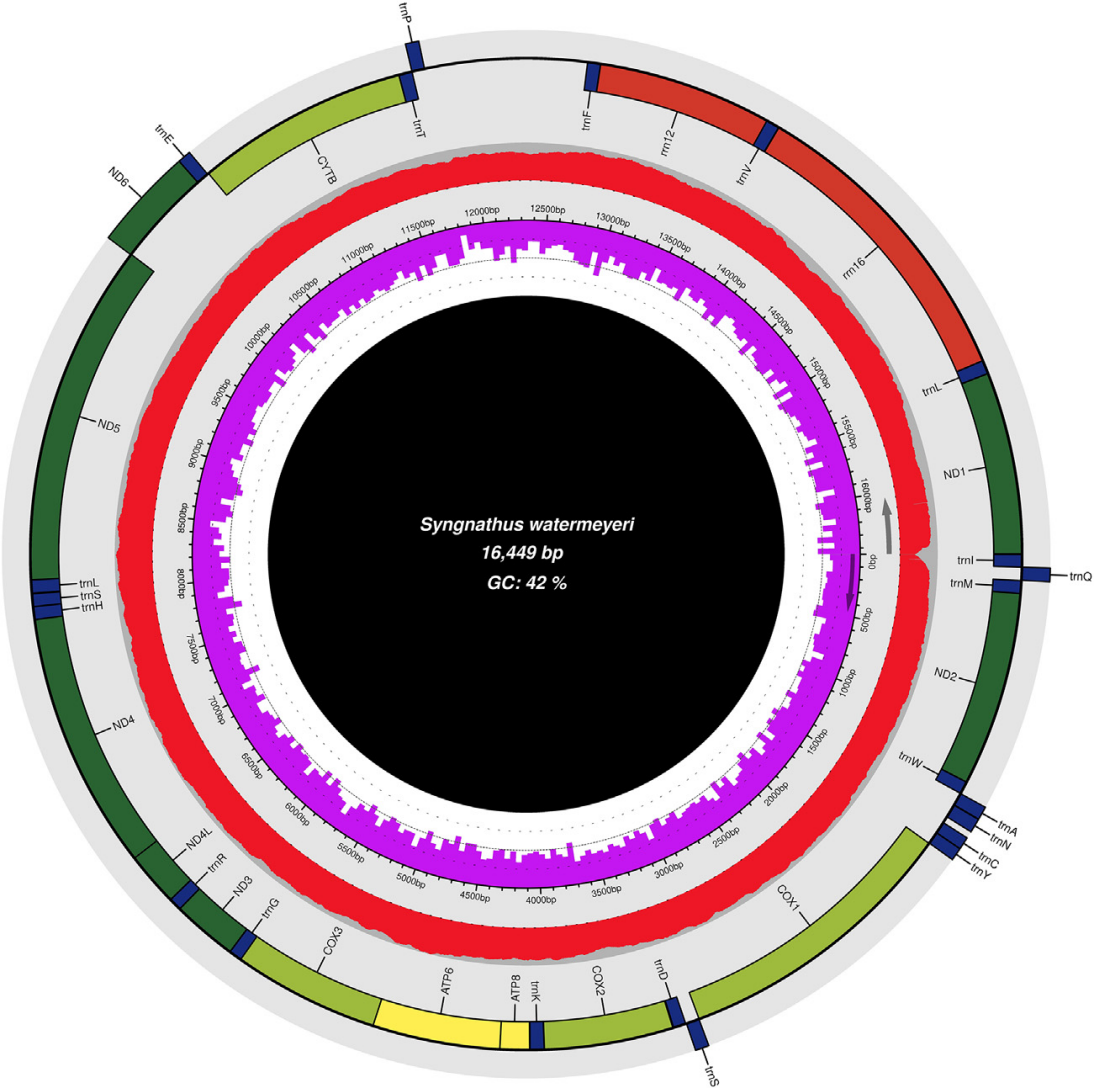
A graphical representation of the *Syngnathus watermeyeri* mitogenome showing the location of 13 protein-coding genes (PCGs), 22 tRNAs, and two rRNAs. The red circle shows the sequence coverage map across the mitogenome, and the purple bars represent the GC content. The graph was generated using Chloroplot [25].

An Illumina NovaSeq 6000 sequencing run yielded a total of 26 459 929 million paired-end (2 × 150 bp) sequences, with an average GC content of 45 %. A two-step denovo mitogenome assembly pipeline reconstructed a consensus circular contig 16 449 bp in length (NCBI accession number OR496150, BioProject PRJNA1007887, and SRA accession number SRR25722163). The nucleotide composition of the assembled mitogenome was estimated as *A* = 29 % (*n* = 4 789), *T* = 29 % (*n* = 4708), *G* = 15 % (*n* = 2500) and C = 27 % (*n* = 4453). Thirteen protein-coding genes (PCGs), 22 tRNAs, and two rRNAs were annotated, as is typical of vertebrate mitochondria [6] (Table 1 and Fig. 2). All the PCGs started with a canonical ATG start codon, the only exception being the *cox1* gene, which had GTG as the start codon. The stop codons TAA and TAG were the most common, but four instances of truncated stop codons were annotated for PCGs, which were T (*cox2*), TA (*cox3*), T (*nad4*) and T (*cytb*). Similar truncated stop codons have been reported for other, closely related pipefish species [7].

**Table 1:** A description of the 37 annotated mitogenomic features in *Syngnathus watermeyeri.*

Feature	Start	End	Strand	Product
tRNA	1	68	+	tRNA-Ile
tRNA	69	138	–	tRNA-Gln
tRNA	140	208	+	tRNA-Met
CDS	209	1250	+	NADH dehydrogenase subunit 2
tRNA	1251	1318	+	tRNA-Trp
tRNA	1319	1387	–	tRNA-Ala
tRNA	1389	1461	–	tRNA-Asn
tRNA	1497	1561	–	tRNA-Cys
tRNA	1562	1628	–	tRNA-Tyr
CDS	1630	3180	+	cytochrome c oxidase subunit I
tRNA	3181	3251	–	tRNA-Ser
tRNA	3255	3322	+	tRNA-Asp
CDS	3326	4016	+	cytochrome c oxidase subunit II
tRNA	4017	4092	+	tRNA-Lys
CDS	4094	4261	+	ATP synthase F0 subunit 8
CDS	4252	4935	+	ATP synthase F0 subunit 6
CDS	4935	5719	+	cytochrome c oxidase subunit III
tRNA	5719	5788	+	tRNA-Gly
CDS	5789	6137	+	NADH dehydrogenase subunit 3
tRNA	6138	6206	+	tRNA-Arg
CDS	6207	6503	+	NADH dehydrogenase subunit 4l
CDS	6497	7877	+	NADH dehydrogenase subunit 4
tRNA	7878	7946	+	tRNA-His
tRNA	7947	8014	+	tRNA-Ser
tRNA	8018	8088	+	tRNA-Leu
CDS	8089	9924	+	NADH dehydrogenase subunit 5
CDS	9921	10,442	–	NADH dehydrogenase subunit 6
tRNA	10,443	10,510	–	tRNA-Glu
CDS	10,516	11,656	+	cytochrome b
tRNA	11,657	11,728	+	tRNA-Thr
tRNA	11,728	11,796	–	tRNA-Pro
tRNA	12,662	12,731	+	tRNA-Phe
rRNA	12,732	13,664	+	12S ribosomal RNA
tRNA	13,664	13,735	+	tRNA-Val
rRNA	13,737	15,399	+	16S ribosomal RNA
tRNA	15,400	15,472	+	tRNA-Leu
CDS	15,473	16,447	+	NADH dehydrogenase subunit 1

**Fig. 3:**
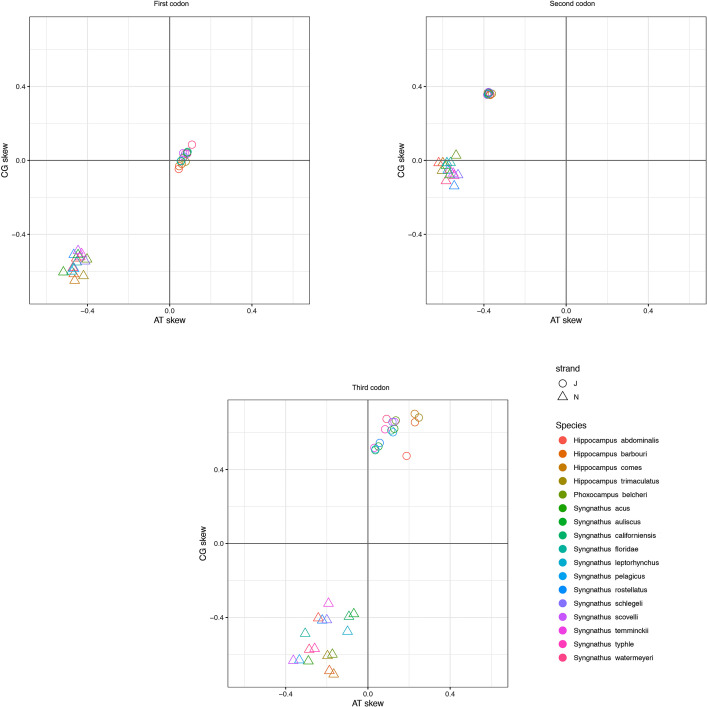
The ratio of AT to CG for each codon estimated for J (light) and N (heavy) strands of the *Syngnathus watermeyeri* mitogenome compared to those estimated for other taxa in this study.

**Fig. 4:**
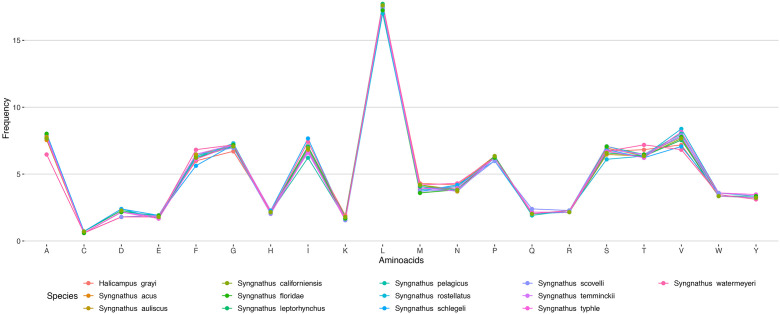
Amino acid frequencies in the complete mitogenome of *Syngnathus watermeyeri* compared to other syngnathids.

**Fig. 5:**
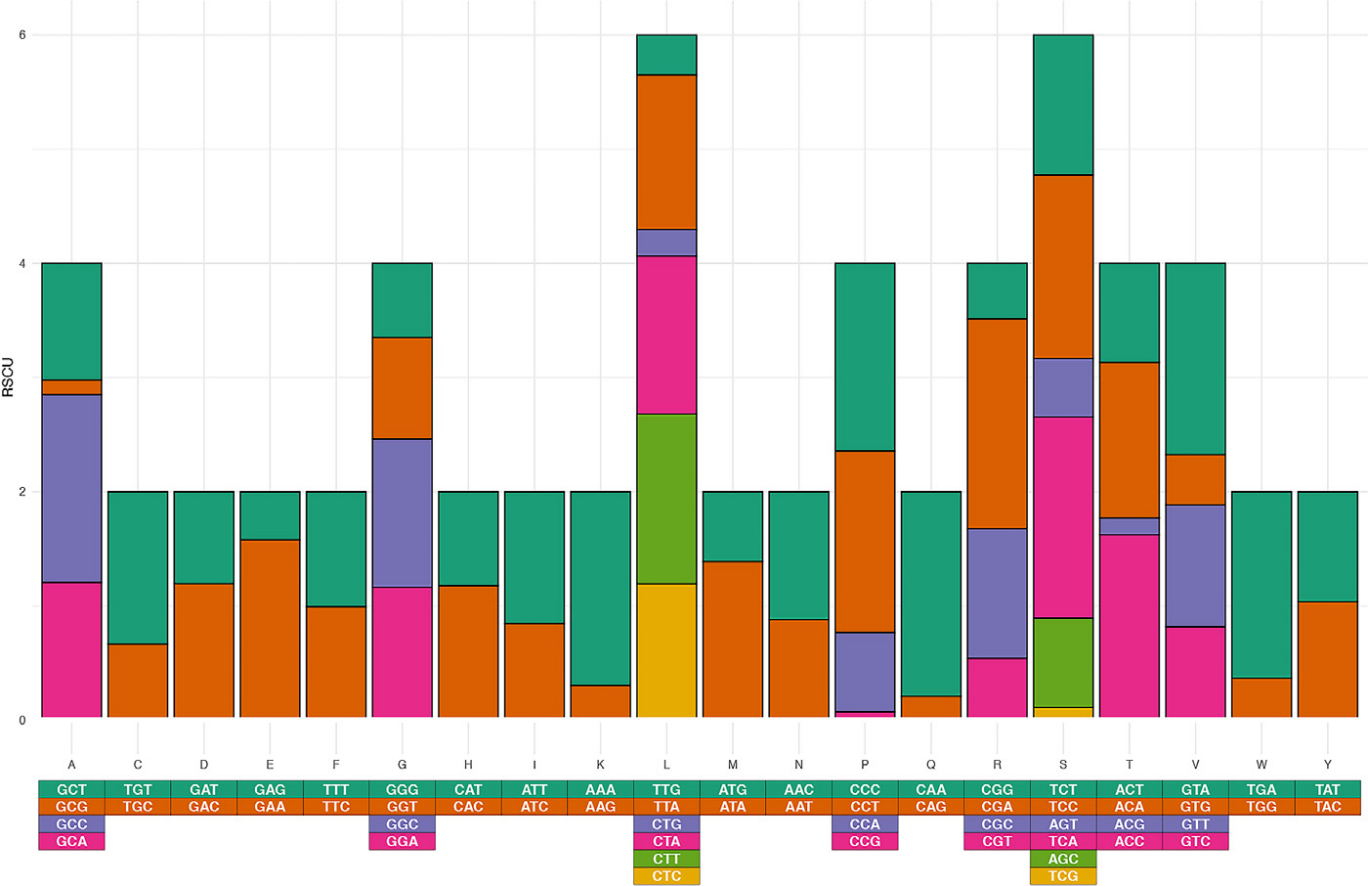
The estimated Relative Synonymous Codon Usage from 13 protein-coding genes in the *S. watermeyeri* mitogenome. Values on the x-axis represent different amino acids.

Similar to the arrangement of PCGs in other species of the pipefish genus *Syngnathus*, short overlaps were identified between atp6 and at8 (9 bp) and between *nad4* and *nad4l* (6 bp) [8,9]. The average distance between the 13 PCGs was 135 bp (range: 1 to 1076 bp) (Supplementary Information, Table 1). The ratio of AT to CG skew for the first, second and third positions of the PCGs, the synonymous codon usage, and the estimated amino acid frequencies (Figs. 3–Fig. 4, Fig. 5, respectively), were consistent with those reported from other syngnathids, confirming that the annotation is complete.

The longest fragment of a non-coding DNA, approximately 866 bp in length, was identified between tRNA-Pro-and tRNA-Phe. When this non-coding sequence was blast-searched against the complete mitogenomes of the other syngnathids included in the study, it showed high similarity to putative control region (CR) annotations (Supplementary Information, Table 2).

**Table 2:** Scientific names and NCBI accession numbers of the taxa that were used for comparative phylogenetics in the study.

Species name	NCBI accession number
*Hippocampus abdominalis*	NC 028181
*Hippocampus barbouri*	NC 024536
*Hippocampus comes*	NC 020336
*Hippocampus trimaculatus*	NC 021107
*Phoxocampus belcheri*	NC 065495
*Syngnathus acus*	MN122937
*Syngnathus auliscus*	OL334979
*Syngnathus californiensis*	NC 063774
*Syngnathus floridae*	NC 065497
*Syngnathus leptorhynchus*	NC 063777
*Syngnathus pelagicus*	NC 065498
*Syngnathus rostellatus*	MN122827
*Syngnathus schlegeli*	NC 037520
*Syngnathus scovelli*	NC 065499
*Syngnathus temminckii*	OR416872
*Syngnathus typhle*	MN122884
*Syngnathus watermeyeri*	OR496150

After removing highly divergent sections of the alignments, the average numbers of phylogenetically informative variable sites in the PCGs of *S. watermeyeri* and the complete alignment, including all other taxa in the study, were 82.30 (SD ± 54.8) and 348.8 (SD± 216.1), respectively. When the complete mitogenome was used, the number of phylogenetically informative sites increased to an average of 1831 ± 54.8 sites per mitogenome and a total of 5747 sites for the full alignment.

**Fig. 6:**
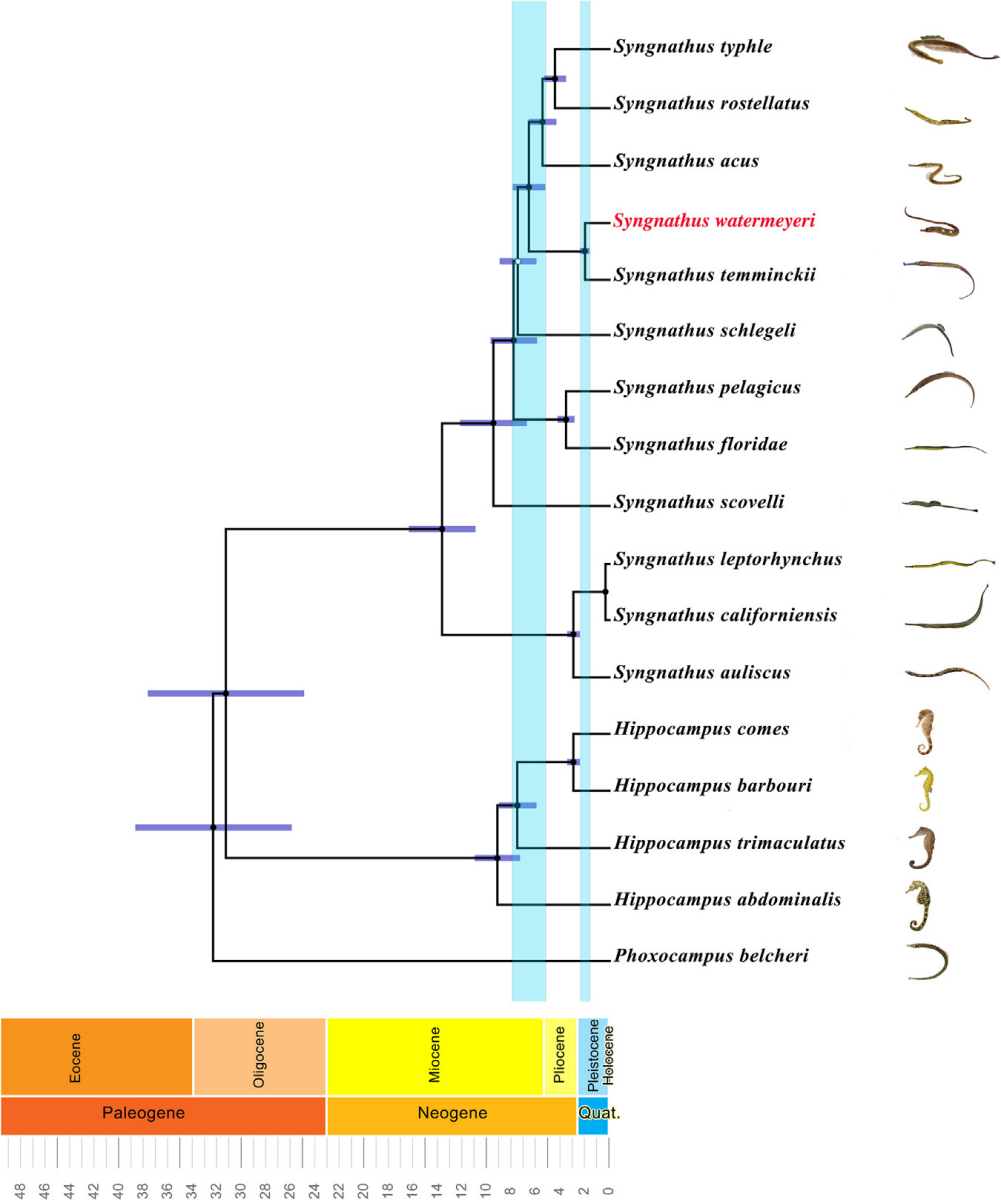
A Bayesian phylogenetic tree showing the phylogenetic placement and the estimated divergence time of *Syngnathus watermeyeri* compared to the other taxa in this study. Black and white nodes correspond to Highest Posterior Density (HPD) intervals greater than 95 % and between 70 and 95 %, respectively. Time is shown in millions of years. The light blue vertical bars depict the divergence times and 95 % HPDs between the southern and northern hemisphere pipefish clades (left strip) and that between the two southen African pipefish species (right strip).

Reconstrutions of evolutionay relationships using maximum likelihood and two Bayesian methods produced phylogenetic trees with identical topologies (Fig. 6 and Supplementary Information Fig. 1). The inspection of trace files of the Bayesian phylogenetic analysis in Tracer v.1.7 [10] confirmed that all replicates reached convergence, with an Effective Sample Size (ESS) greater than 1000.

In the reconstructed mitogenomic tree, *Syngnathus watermeyeri* was recovered as the sister taxon of its widely distributed southern African congener, *S. temminckii*. The two southern African species formed a monophyletic group with a clade of northern hemisphere pipefish that comprises *S. acus, S. rostellatus,* and *S. typhle.* The split between the southern and northern hemisphere clades, and that between the two endemic southern African species, was estimated at 5.3 Ma (95 % HPD: 4.6–6.1 Ma) and 1.5 Ma (95 % HPD: 1.2–1.7 Ma), respectively (see Table 2 for the list of species used for the phylogenetic analysis and Fig. 6).

The estimated mitogenomic divergence between the northern and southern hemisphere clades of pipefish coincides with the end of an episode in the Miocene when the family Syngnathidae experienced exceptionally high diversification rates across the globe. During this period, seahorses (genus *Hippocampus*) in the Central Indo-Pacific, and pipefishes of the genus *Syngnathus* in the Western Atlantic, diverged into a number of new evolutionary lineages [11]. The exact cause of this phenomenon remains unknown, but it has been suggested that during the Middle Miocene Climate Transition (14.5–12.5 Ma), when net diversification rates were at their highest, the latitudinal sea level surface temperature gradients that prevented marine species from expanding their ranges, temporarily disappeared for millions of years [12]. Consequently, some Atlantic species may have expanded their ranges into new habitats, including southern Africa. Following subsequent temporal and spatial isolation, these peripheral populations evolved into new species. The divergence between the two endemic southern African species was estimated to have occurrred during the Pleistocene. The driver of this speciation event, and its consequences for the evolutionary potential of Critically Endangered estuarine pipefish, is currently a topic of active research.

## 4 Experimental Design, Materials and Methods

A specimen of *S. watermeyeri* was caught in the Kariega Estuary, South Africa (−33.67378 south, 26.67484 east) using a seine net. A fin clip from the tail fin (∼1.5 mm^2^) was collected using a pair of sterile cuticle scissors, and the fish was immediately released back into the same submerged seagrass habitat where it had been capturerd. The excised tissue was preserved in 99 % ethanol. Genomic DNA was extracted using the Qiagen DNeasy Blood & Tissue Kit (Qiagen, Hilden, Germany) within 24 h of collection. The genomic DNA was sheared into shorter fragments using the Diagenode Bioruptor (Diagenode, New Jersey, United States), end-repaired, and adapter-ligated using the NOVOKit (Novogene, Beijing, PRC). A subset of fragments ∼350 bp in length were selected for the amplification step. Prior to sequencing, the quality of the constructed genomic library was checked using Qubit (Thermo Fisher Scientific, Waltham, USA), qPCR, and the DNA NGS 3K assay (PerkinElmer, Waltham, USA), and subsequently, the genomic library was sequenced on the NovaSeq 6000 SP platform (Illumina, San Diego, USA) using paired-end 150 bp chemistry following the standard Illumina protocol.

The mitogenome was assembled using a two-step method. First, the complete mitogenome was assembled denovo by direct extension of a starting *cytochrome b* sequence from the same species (NCBI accession number JX228139.1) using NOVOPlasty v4.3 [13]. The assembled circular contig was then used as the guiding reference template in the second step to re-assemble the mitogenome in GetOrganelle v1.7 [14]. In both assembly methods, the assembly settings were set to their default values. The consensus mitogenome was annotated using a combination of the MITOS web server [15] and the “annotate” subcommand in MitoZ v3.6 [16], and the boundaries of annotated features were visually adjusted in Geneious Prime v.2023.0 (Dotmatics, Boston, MA) based on homologous sections of the *Syngnathus acus* mitogenome (NCBI accession: MN122937.1).

Codon skew [17], amino acid frequencies, and Relative Synonymous Codon Usage (RSCU) for each protein-coding gene were calculated in EZmito (Cucini et al., 2021). The evolutionary history of *S. watermeyeri* was reconstructed using 13 protein-coding sequences from 16 closely related species of the teleost fish family Syngnathidae (12 pipefish species and four seahorses) (Table 1). These were identified based on blast search results, and were downloaded from the NCBI database.

Phylogenetic relationships between the selected taxa were reconstructed using Maximum Likelihood and two methods of Bayesian Inference. A consensus Maximum Likelihood (ML) phylogenetic tree was reconstructed using IQ-TREE2 v.2.2 [18] using 9999 bootstrap replications. To select the best partitioning scheme for each protein-coding gene, the greedy search implemented in ModelFinder as part of the same pipeline was used [19]. A Bayesian phylogenetic tree was reconstructed in BEAST2 v2.5 [20] using the Bayesian phylogenetic site model averaging package, bModelTest, as part of the same package. In this method, the uncertainty in the nucleotide substitution model and the tree topology are simultaneously simulated. In the second Bayesian method, the phylogenetic relationships between the same group of taxa was reconstructed based on the complete mitogenome in the auto-partition analysis implemented in the Reversible-Jump Based (RB) package [21], which is implemented in BEAST package. In the auto partition method, the complete mitogenome is divided into an arbitrary number of partitions, and the tree topology, the border of each partition, and the best nucleotide substitution model for each partition are simultaneously simulated. To estimate the approximate divergence time between *S. watermeyeri* and a subset of its closely related congeners from the southern and northern hemispheres, prior distributions for the Time to the Most Recent Common Ancestor (TMRCA) that correspond to the divergence between all members of the subfamily Syngnathini (lognormal, mean = 12.89 in real space), that of all Hippocampinae (normal, mean = 13.37, S.D. = 2.5), and the prior for the split between Syngnathidae and Hippocampinae (normal, mean = 36.1, S.D. = 3.2) were set based on the estimates for the same group of taxa in other studies that were inferred from a combination of fossil and molecular evidence [11,22,23]. Among different molecular clock models in BEAST, a strict molecular clock was selected, and the reaming parameters in both analyses were set to their default values. BEASTwas run for ten independent replicates, each 200 million iterations long with an initial 50 million burn-in steps. The resulting tree was visualised using a combination of Figtreev1.4 (https://github.com/rambaut/figtree) and strap v.1.6 [24]

## Limitations

None.

## Ethics Statement

The research permit for this study (RES2020/101) was granted by the Department of Forestry, Fisheries and the Environment (DFFE) of the Republic of South Africa in accordance with IUCN requirements. The animal ethics clearance for this study was approved by the Faculty of Science Ethics Committee at the University of Johannesburg (Ethics Reference Number: 2020–02–06/Teske_Weiss).

## CRediT Author Statement

Not Applicable to a single-authored data note.

## Data Availability

NCBI accession number (Original data) (NCBI) NCBI Raw data (Original data) (NCBI) NCBI Raw data (Original data) (NCBI) NCBI Raw data (Original data) (NCBI) NCBI Raw data (Original data) (NCBI)

## Appendix Supplementary materials

Image, application 1

Image, application 2
