# Supplementary material for: The complete mitogenome dataset of the Critically Endangered estuarine pipefish, Syngnathus watermeyeri
Source: Data Brief. 2023 Nov 29;52:109864. doi: 10.1016/j.dib.2023.109864 (PMC10749243; doi:10.1016/j.dib.2023.109864)
Supplement: Supplementary file 1 [file mmc1.docx]

**Table 1** The intergenic distance between 13 Protein-coding genes in the Sygnathus watermeyeri mitogenome. The negative values show the size of overlap between the two protein-coding genes.The snippet point is shown by a (+).

| Feature | Start | End | Strand | Product | The intergenic distance with the next mitogenomic feature (bp) |
| --- | --- | --- | --- | --- | --- |
| CDS | 209 | 1250 | + | NADH dehydrogenase subunit 2 | 380 |
| CDS | 1630 | 3180 | + | cytochrome c oxidase subunit I | 146 |
| CDS | 3326 | 4016 | + | cytochrome c oxidase subunit II | 78 |
| CDS | 4094 | 4261 | + | ATP synthase F0 subunit 8 | -9 |
| CDS | 4252 | 4935 | + | ATP synthase F0 subunit 6 | 0 |
| CDS | 4935 | 5719 | + | cytochrome c oxidase subunit III | 70 |
| CDS | 5789 | 6137 | + | NADH dehydrogenase subunit 3 | 70 |
| CDS | 6207 | 6503 | + | NADH dehydrogenase subunit 4L | -6 |
| CDS | 6497 | 7877 | + | NADH dehydrogenase subunit 4 | 212 |
| CDS | 8089 | 9924 | + | NADH dehydrogenase subunit 5 | -3 |
| CDS | 9921 | 10442 | - | NADH dehydrogenase subunit 6 | 74 |
| CDS | 10516 | 11656 | + | cytochrome b | 1076 |
| rRNA | 12732 | 13664 | + | 12S ribosomal RNA | 73 |
| rRNA | 13737 | 15399 | + | 16S ribosomal RNA | 74 |
| CDS | 15473 | 16447 | + | NADH dehydrogenase subunit ^+^ | 211 |

**Table 2** The genetic distance between all 37 features in the Sygnathus watermeyeri mitogenome. The negative values show the size of overlap between the two features. The snippet point is shown by a (+).

| Feature | Start | End | Strand | Product | The diststance with the next mitogenomic feature (bp) |
| --- | --- | --- | --- | --- | --- |
| tRNA | 1 | 68 | + | tRNA-Ile | 1 |
| tRNA | 69 | 138 | - | tRNA-Gln | 2 |
| tRNA | 140 | 208 | + | tRNA-Met | 1 |
| CDS | 209 | 1250 | + | NADH dehydrogenase subunit 2 | 1 |
| tRNA | 1251 | 1318 | + | tRNA-Trp | 1 |
| tRNA | 1319 | 1387 | - | tRNA-Ala | 2 |
| tRNA | 1389 | 1461 | - | tRNA-Asn | 36 |
| tRNA | 1497 | 1561 | - | tRNA-Cys | 1 |
| tRNA | 1562 | 1628 | - | tRNA-Tyr | 2 |
| CDS | 1630 | 3180 | + | cytochrome c oxidase subunit I | 1 |
| tRNA | 3181 | 3251 | - | tRNA-Ser | 4 |
| tRNA | 3255 | 3322 | + | tRNA-Asp | 4 |
| CDS | 3326 | 4016 | + | cytochrome c oxidase subunit II | 1 |
| tRNA | 4017 | 4092 | + | tRNA-Lys | 2 |
| CDS | 4094 | 4261 | + | ATP synthase F0 subunit 8 | -9 |
| CDS | 4252 | 4935 | + | ATP synthase F0 subunit 6 | 0 |
| CDS | 4935 | 5719 | + | cytochrome c oxidase subunit III | 0 |
| tRNA | 5719 | 5788 | + | tRNA-Gly | 1 |
| CDS | 5789 | 6137 | + | NADH dehydrogenase subunit 3 | 1 |
| tRNA | 6138 | 6206 | + | tRNA-Arg | 1 |
| CDS | 6207 | 6503 | + | NADH dehydrogenase subunit 4L | -6 |
| CDS | 6497 | 7877 | + | NADH dehydrogenase subunit 4 | 1 |
| tRNA | 7878 | 7946 | + | tRNA-His | 1 |
| tRNA | 7947 | 8014 | + | tRNA-Ser | 4 |
| tRNA | 8018 | 8088 | + | tRNA-Leu | 1 |
| CDS | 8089 | 9924 | + | NADH dehydrogenase subunit 5 | -3 |
| CDS | 9921 | 10442 | - | NADH dehydrogenase subunit 6 | 1 |
| tRNA | 10443 | 10510 | - | tRNA-Glu | 6 |
| CDS | 10516 | 11656 | + | cytochrome b | 1 |
| tRNA | 11657 | 11728 | + | tRNA-Thr | 0 |
| tRNA | 11728 | 11796 | - | tRNA-Pro | 866 |
| tRNA | 12662 | 12731 | + | tRNA-Phe | 1 |
| rRNA | 12732 | 13664 | + | 12S ribosomal RNA | 0 |
| tRNA | 13664 | 13735 | + | tRNA-Val | 2 |
| rRNA | 13737 | 15399 | + | 16S ribosomal RNA | 1 |
| tRNA | 15400 | 15472 | + | tRNA-Leu | 1 |
| CDS | 15473 | 16447 | + | NADH dehydrogenase subunit ^+^ | 2 |


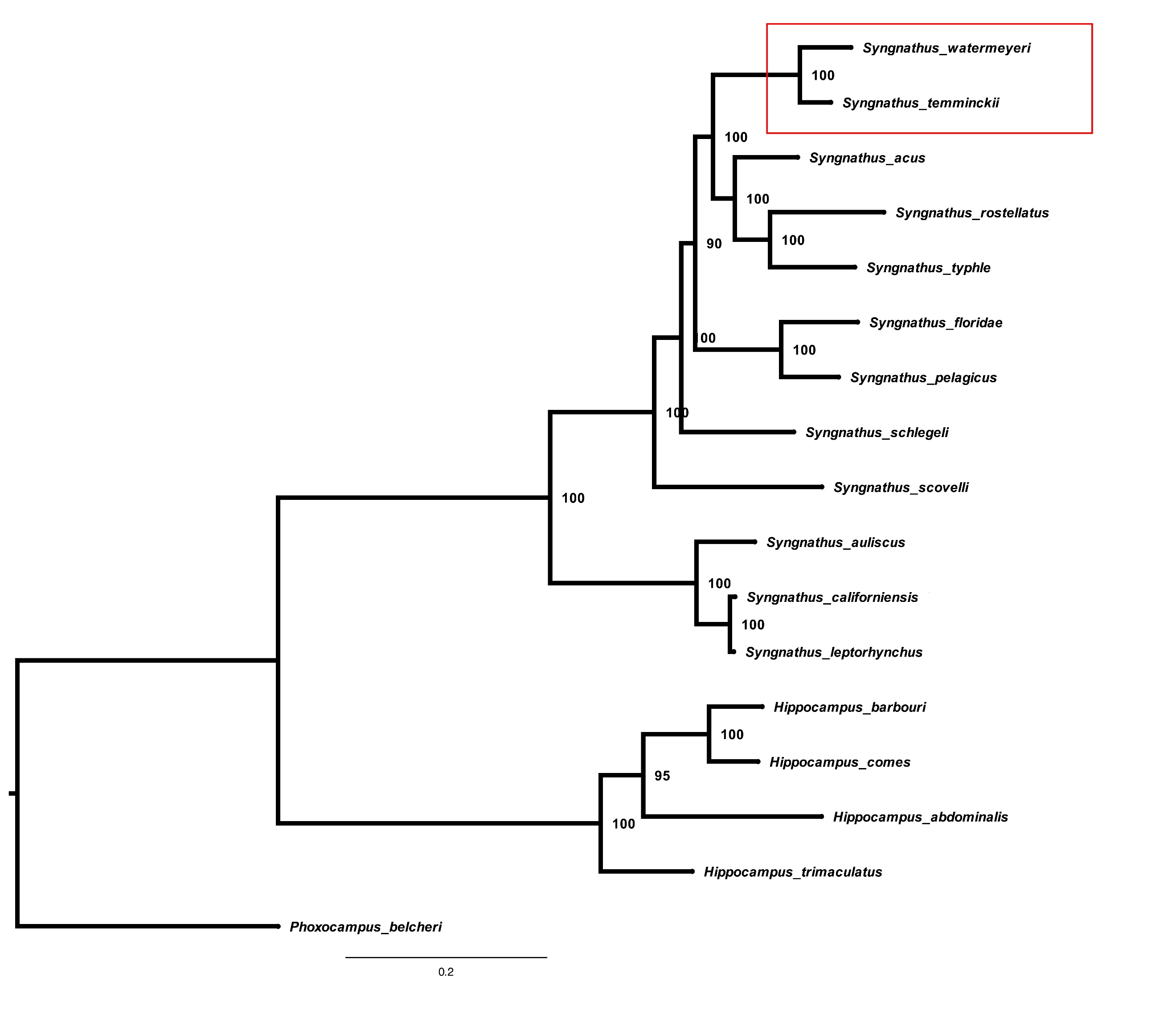


**Figure 1** A Maximum Likelihood phylogenetic tree showing the phylogenetic status of *Syngnathus watermeyeri* compared to the other taxa in this study. Numbers show bootstrap support. The red rectangle shows the clade of endemic South African pipefish.
